# Isorhamnetin inhibits migration and promotes apoptosis via modulation of oxidative stress, mitochondrial dysfunction, and the TIGIT/CD155 axis in 4T1 breast cancer cells

**DOI:** 10.3389/fcell.2026.1829358

**Published:** 2026-05-07

**Authors:** Fei Chen, Yang Liu, Hong Zhang, Qinglin Yang, Guoxin Li, Ying Zhu, Rui Zhang

**Affiliations:** 1 Medical Laboratory Centre, The Third Affiliated Hospital of Jinzhou Medical University, Jinzhou, China; 2 Department of General Surgery, The Third Affiliated Hospital of Jinzhou Medical University, Jinzhou, China

**Keywords:** 4T1, breast cancer, isorhamnetin, migration, mitochondrial dysfunction, oxidative stress, TIGIT/CD155

## Abstract

**Background:**

Isorhamnetin (ISO), a natural flavonoid derived from traditional Chinese medicine, has demonstrated potential antitumor activity. This study aimed to investigate the effects of ISO on the proliferation, migration, and apoptosis of mouse breast cancer 4T1 cells and to explore the underlying mechanisms.

**Methods:**

Cell viability was evaluated using a Cell Counting Kit-8 (CCK-8) assay. Wound healing and colony formation assays were performed to assess migration and clonogenic ability. Intracellular reactive oxygen species (ROS) levels and mitochondrial membrane potential were measured to evaluate oxidative stress and mitochondrial function. Quantitative polymerase chain reaction and Western blot analyses were conducted to determine the expression of the TIGIT/CD155 signaling axis.

**Results:**

ISO inhibited 4T1 cell proliferation in a concentration- and time-dependent manner. It significantly reduced migration and clonogenic capacity. ISO treatment increased intracellular ROS levels and disrupted mitochondrial membrane potential, indicating oxidative stress–mediated mitochondrial dysfunction. Additionally, ISO markedly downregulated the mRNA and protein expression levels of the TIGIT/CD155 signaling axis (p < 0.01).

**Conclusion:**

ISO suppresses proliferation and migration and induces apoptosis in 4T1 breast cancer cells, potentially through modulation of oxidative stress, mitochondrial dysfunction, and the TIGIT/CD155 signaling axis. These findings provide mechanistic insight into the antitumor effects of ISO and support its potential as a therapeutic candidate for breast cancer.

## 1 Introduction

Breast cancer is one of the most common cancers among women. It remains the second leading cause of cancer-related death in women worldwide despite decades of advances in early detection and treatment that have reduced mortality rates. Moreover, the increasing trends of delayed childbirth, reduced fertility, and irregular lifestyles have led to an annual rise in the incidence of breast cancer in women (Giaquinto et al., 2024). Studies have shown (Miglietta et al., 2022) a significant progress in the treatment of metastatic breast cancer in recent years, particularly in extending progression-free survival (PFS) and overall survival (OS) in some patients. However, cancer metastasis involves complex pathophysiological processes such as angiogenesis, epithelial–mesenchymal transition (EMT), and regulation of tumor metastasis–related gene expression (Fedele et al., 2017; Wang et al., 2018; Lian et al., 2025). Therefore, new therapeutic strategies are urgently needed to prevent further metastasis and progression of breast cancer. Traditional Chinese medicine (TCM) has attracted widespread attention in recent years as a complementary approach to cancer immunotherapy. Research on its ability to enhance the body’s antitumor capacity by regulating the immune system has become a key focus (Lei and Chen, 2025). Studies have indicated that the combination of TCM and immune checkpoint inhibitors may exert synergistic effects (Tong et al., 2025). The repurposing of more active ingredients from TCM has emerged as an alternative strategy for anticancer drug development, thereby increasing treatment options for patients with metastatic tumors.

Isorhamnetin (ISO) is a flavonoid compound isolated and purified from the fruits of *Hippophae rhamnoides* L. (a plant belonging to the Elaeagnaceae family). It possesses medicinal effects such as relieving cough and phlegm, promoting blood circulation to resolve stasis, and aiding digestion. It is commonly used in the treatment of excessive cough and phlegm, indigestion, and so forth. Recent studies have found that ISO, as a common natural active ingredient in TCM, exhibits potent antitumor activity. Its mechanism involves regulating the expression of cell cycle–related proteins (e.g., CyclinB, CyclinD, CyclinE, and CyclinA) and increasing the proportion of cells in the S phase, thereby inhibiting tumor cell proliferation (Li et al., 2021). Additionally, it can activate the PI3K/Akt signaling pathway to promote the expression of proliferation-related proteins (e.g., PCNA and c-myc) (Li et al., 2022), and inhibit the mTOR pathway to further restrict tumor cell survival and proliferation (Biswas et al., 2024). In terms of improving the tumor microenvironment, bifunctional nanoparticles co-loaded with ISO and anti-PD-L1 antibodies can reduce the proportion of myeloid-derived suppressor cells (MDSCs) in the tumor microenvironment while promoting T cell infiltration. This effect may be achieved by inhibiting USP7-mediated deubiquitination of YY1, thereby providing a new strategy for treating hepatocellular carcinoma (Liu et al., 2023). However, reports on the effect and mechanism of ISO on breast cancer metastasis are few.

T cell immunoreceptor with Ig and ITIM domains (TIGIT) and its ligand CD155 have received extensive attention in recent years as emerging immune checkpoints. CD155 is the primary ligand of TIGIT and exerts co-inhibitory or co-stimulatory effects in the tumor microenvironment by binding to receptors such as TIGIT, CD96, and CD226 (Zhou et al., 2023). Studies have shown that (Huang et al., 2024) the TIGIT/CD155 interaction inhibits CD8^+^ T cell function, characterized by reduced secretion of cytokines (e.g., IFN-γ and TNF-α), and influences T cell metabolism and activation by inhibiting the PI3K/Akt/mTOR and NF-κB/ERK signaling pathways. Furthermore, TIGIT/CD155 signaling impairs the immunosurveillance function of NK cells, and blocking this pathway can restore NK cell-mediated killing of circulating tumor cells (CTCs) (Sun et al., 2025). In addition to their well-known roles in immune regulation, immune checkpoint molecules like TIGIT and CD155 have also been recognized for their potential functions within tumor cells. Recent studies suggest that (Zheng et al., 2023; Liu et al., 2021) this axis may be expressed in tumor cells and is associated with malignant phenotypes, such as enhanced cell proliferation, migration, and survival. Despite these findings, the role of TIGIT/CD155 signaling in breast cancer cells, particularly in response to metabolic stress and natural compound treatment, remains under investigation.

In this study, we investigated the effects of ISO on the proliferation, migration, invasion, and apoptosis of 4T1 breast cancer cells. Our results suggest that ISO inhibited migration and promoted apoptosis in metastatic breast cancer cells. Based on these findings, we hypothesize that ISO may exert its effects on 4T1 cells through the TIGIT/CD155 signaling axis, which could be involved in regulating breast cancer cell proliferation, migration, and metastasis in response to ISO treatment.

## 2 Materials and methods

### 2.1 Cell culture and treatment

The 4T1 mouse mammary carcinoma cell line and HC11 mouse thymic epithelial cell line were both purchased from KeyGEN BioTECH Co., Ltd., (Nanjing, China). ISO (purity ≥ 98%, Cat number: SI8280) was purchased from Solarbio Science and Technology Co., Ltd., (Beijing, China).

The 4T1 and HC11 cell lines were maintained in standard T75 cell culture flasks (NEST, WuXi, China) at 37 °C in a humidified incubator with 5% CO_2_. They were grown in Dulbecco’s modified Eagle’s medium (Keycell Biotechnology, Wuhan, China) supplemented with 10% fetal bovine serum (Keycell Biotechnology) and 1% penicillin/streptomycin (Keycell Biotechnology).

The cells were passaged when the cell density reached 80%. Initially, the existing medium was discarded and the cells were washed once with phosphate-buffered saline (PBS). Subsequently, 1 mL of trypsin (Keycell Biotechnology) was added to facilitate cell digestion. The cells were observed under a microscope. They detached and became rounded after 2 min of digestion, signaling that digestion was complete. Trypsin was immediately removed, complete medium was added, and the cells were gently pipetted to create a single-cell suspension. The cells were passaged at a 1:3 ratio and continuously cultured at 37 °C in a 5% CO2 atmosphere with saturated humidity. All assays were performed with cells in a good logarithmic growth phase.

### 2.2 Cell proliferation assay

Cell viability was analyzed using the Cell Counting Kit-8 (CCK-8). 4T1 and HC11 cells were seeded at a density of 1 × 10^3^ cells per well in a 96-well plate (100 µL per well). Various concentrations of ISO (0, 0.1, 1, 3, 6, 12.5, 25, 50, 100, and 200 µM) were added, and the cells were incubated for 12, 24, 48, and 72 h. Subsequently, 100 µL of CCK-8 reagent (Keycell Biotechnology) was added to each well and incubated at 37 °C for 2 h. The absorbance values at 450 nm were then measured using a microplate reader. The experiment was repeated at least three times.

### 2.3 Wound healing assay

4T1 cells in the logarithmic growth phase and growing well were seeded at a density of 5 × 10^5^ cells per well in a six-well plate. They were incubated overnight at 37 °C in an incubator with 5% CO_2_. The cells were treated with different concentrations of ISO (0, 6, 12.5, and 25 μM) for 48 h. The cells were then digested with trypsin and collected. They were seeded at a density of 1 × 10^6^ cells per well in a six-well plate and cultured overnight at 37 °C under 5% CO_2_ and saturated humidity. Once the cell density exceeded 90%, a wound was created in the center of each well using the tip of a sterile 200-μL pipette. Cell debris was then washed away with PBS. The images were taken after 0, 12, 24, and 48 h using an inverted microscope. The wound area was measured using ImageJ software. The assay was repeated at least three times.

### 2.4 Colony formation assay

The 4T1 cells were treated with different concentrations of ISO (0, 6, 12.5, and 25 μM) for 48 h. They were then trypsinized and seeded at a density of 300 cells per well in six-well plates. The culture plates were placed at 37 °C in a 5% CO_2_ incubator for 2 weeks. After colony formation, the supernatant was discarded. The cells were washed twice with PBS and then fixed with ice-cold ethanol for 1 h. The fixative was discarded, a sufficient amount of crystal violet stain was added for 20 min, the cells were washed with PBS, and the colonies were counted manually.

### 2.5 Cellular ROS assay

The 4T1 cells were treated with different concentrations of ISO (0, 6, 12.5, and 25 μM) for 48 h and then collected by trypsinization. The cell suspension was centrifuged at 1,200 rpm for 5 min, and the supernatant was removed. The cells were washed once with PBS, then centrifuged again at 1,200 rpm for 5 min. Following the protocol for the cellular reactive oxygen species (ROS) detection kit (DCFH-DA was diluted 1:1,000 in serum-free medium to a final concentration of 10 μM). One milliliter of the diluted DCFH-DA was added to the treated cells. The mixture was incubated at 37 °C for 20 min, with gentle mixing every 3 min. After incubation, the cells were washed three times with serum-free medium. Subsequently, they were centrifuged again at 1,200 rpm for 5 min and the supernatant was removed. The cells were resuspended in PBS, and then the processed cell samples were analyzed using flow cytometry.

### 2.6 Mitochondrial membrane potential assay

The 4T1 cells were treated with different concentrations of ISO (0, 6, 12.5, and 25 μM) for 48 h. They were then digested with trypsin, collected, and centrifuged at 1,200 rpm for 3 min. The supernatant was removed. The cells were resuspended in PBS and washed once. They were again centrifuged at 1,200 rpm for 3 min. Subsequently, they were processed using the mitochondrial membrane potential assay kit (Keycell Biotechnology), followed by analysis via flow cytometry.

### 2.7 Mechanisms of metastasis and apoptosis in 4T1 cells

#### 2.7.1 qPCR assay

4T1 cells in the logarithmic growth phase exhibiting robust growth were seeded at a cell density of 5 × 10^5^ cells per well in a six-well plate and incubated at 37 °C in a 5% CO_2_ incubator for 24 h. They were treated with different concentrations of ISO (0, 6, 12.5, and 25 μM) for 48 h. Total cellular RNA was extracted using TRIzol reagent (Ambion, TX, USA), reverse transcribed into cDNA (Vazyme Biotech, Nanjing, China), and subjected to real-time quantitative fluorescence gene detection (Vazyme Biotech). The qPCR reaction conditions were as follows: 95 °C for 10 min, one cycle; 95 °C for 10 s and 60 °C for 60 s, 40 cycles; 95 °C for 15 s, 60 °C for 60 s, and 95 °C for 15 s, one cycle. GAPDH was employed as the internal reference gene. The sequences of the primers used in this study are provided in Table 1.

**TABLE 1:** Primers used in the study.

Name	Primer	Sequence	Size (bp)
Mus *GAPDH*	Forward	ATG​GGT​GTG​AAC​CAC​GAG​A	229
	Reverse	CAG​GGA​TGA​TGT​TCT​GGG​CA	
Mus *TIGIT*	Forward	TGG​GAC​TCA​TTT​GCT​TAA​TGG​T	164
	Reverse	AGT​TTG​TGT​CTG​GAC​AGG​GCT​T	
Mus *CD155*	Forward	CTG​CTG​TTC​TGC​TAT​GCA​CTC​C	232
	Reverse	CCC​TCT​CTG​GCT​CTT​TGA​TGT​T	

#### 2.7.2 Western blot assay

The 4T1 cells were treated with different concentrations of ISO (0, 6, 12.5, and 25 μM) for 48 h before harvest. Cells were lysed using RIPA lysis buffer (Servicebio Technology, Wuhan, China) supplemented with 100 mM phenylmethylsulfonyl fluoride (PMSF) and 100 mM sodium vanadate, and protein concentrations were determined using a BCA protein quantification kit (GBCBIO Technologies, Guangzhou, China).

Protein samples and prestained molecular weight markers (Cat. No. M00521, GenScript, Nanjing, China) were loaded onto the gel. Electrophoresis was performed at a constant voltage of 80 V until the bromophenol blue indicator migrated to the inter-face between the 5% stacking gel and 12% separating gel as a linear band. The voltage was then increased to 120 V, and electrophoresis continued until the dye front reached the bottom of the gel, with a total duration of approximately 1.5 h. Equal amounts of protein were then transferred to a polyvinylidene fluoride (PVDF) membrane (Millipore, Billerica, MA, USA).

The membrane was blocked with 5% non-fat milk in TBST buffer for 2 h at room temperature with gentle shaking, followed by incubation with primary antibody against TIGIT (Cat. No. 83545-1-RR, RRID: AB_3671164, 1:1,000, Protein tech Group, Inc.) diluted in blocking buffer overnight at 4 °C. After washing, the membrane was incubated with horseradish peroxidase (HRP)-conjugated secondary antibodies for 2 h at room temperature. Finally, the ECL reagent was prepared by mixing the enhanced solution and stable peroxidase solution at a 1:1 ratio and added dropwise onto the PVDF membrane. After incubation for several minutes until clear fluorescent bands appeared, excess substrate solution was removed with filter paper, and the membrane was covered with plastic wrap and exposed to X-ray film. The film was sequentially processed with developer solution and fixer solution (Solarbio Science and Technology Co., Ltd., Beijing, China), washed, air-dried, and scanned. The grayscale values were analyzed using Image-Pro Plus (IPP) software. After TIGIT detection, the PVDF membrane was stripped and sequentially incubated with primary antibodies against CD155/PVR (Cat. No. A00664-3, 1:2000, Wuhan Boster Biological Technology, Ltd.) and GAPDH (Cat. No. 83545-1-RR, RRID: AB_2107436, 1:50,000, Protein tech Group, Inc.) at 4 °C overnight, respectively. After washing and incubation with HRP-conjugated secondary antibody, each protein was detected by ECL, and band intensities were analyzed using Image-Pro Plus software.

### 2.8 Statistical analysis

The data for statistical analysis were obtained from at least three independent experiments for each method. The GraphPad Prism 10.5.0 was used to conduct the statistical analysis. Basic statistical characteristics were computed, and significant differences were determined using one-way analysis of variance (ANOVA). The Shapiro-Wilk normality test was used to evaluate the normality of the variables. ANOVA was followed by Dunnett’s test, which computed a confidence interval for the difference between two means by comparing each mean to a control mean. The differences were compared to assess statistical significance at the levels of *P* < 0.001 (^***^); *P* < 0.01 (^**^), and *P* < 0.05 (^*^).

## 3 Results

### 3.1 ISO inhibited the proliferation of 4T1 cells

**FIGURE 1:**
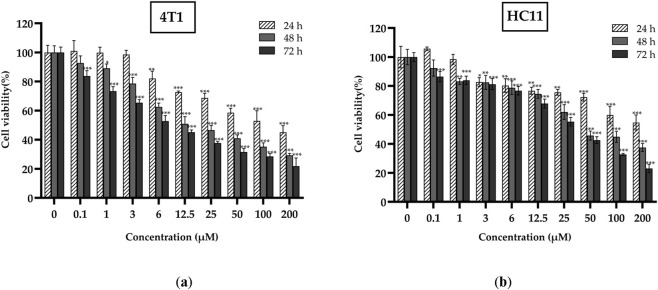
Mouse 4T1 mammary carcinoma cells **(a)** and mouse HC11 mammary epithelial cells **(b)** were seeded into 96-well plates and treated with isorhamnetin (ISO) at indicated concentrations for 24, 48, and 72 h. Cell proliferation was assessed using the CCK-8 assay. Data are presented as mean ± SD from three independent experiments (n = 3). Statistical analysis was performed using one-way ANOVA. *p < 0.05, **p < 0.01, ***p < 0.001 vs. control group.

The 4T1 cells were treated with different concentrations of ISO, and cell viability was assessed using the CCK-8 assay. The results are presented in Figure 1a. For a 24 h treatment duration, low concentrations (0–3 µM) of ISO exhibited no significant inhibitory effect on 4T1 cells. However, at concentrations of 6 µM and above, regardless of exposure duration (24, 48, or 72 h), ISO exhibited significant or highly significant inhibition of cell proliferation, with viability consistently below 70%. This inhibitory effect demonstrated a pronounced concentration- and time-dependent relationship.

As a control group, HC11 cells exhibited a distinct pattern of viability changes compared with 4T1 cells, as shown in Figure 1b. Starting with a treatment concentration of 1.0 µM, ISO demonstrated a slight inhibitory effect, yet cell viability remained at approximately 80%. This inhibitory effect intensified over time, although a significant reduction in viability occurred only at the high concentration of 25 µM (48–72 h). Nevertheless, the extent of this decline was markedly lower than that observed in 4T1 cells treated under identical conditions.

In summary, ISO inhibited the proliferation of 4T1 cells, depending on concentration and treatment duration. The IC50 of ISO in 4T1 cells at 48 h was determined as 15.871 µM. When the ISO concentration ranged from 6 to 25 µM, the cell viability at 48 h was significantly inhibited compared with that at 24 and 72 h, with the cell viability ranging between 50% and 70% at this time. When the concentration exceeded 25 µM, the cell viability at both 48 h and 72 h was lower than 50%. The viability of HC11 cells remained at approximately 80% when the ISO concentration was 12.5 µM. Although high concentrations and prolonged treatment inhibited HC11 cell viability, the inhibition magnitude was much lower than that on 4T1 cells under the same conditions. These concentrations represent low, moderate, and high levels relative to the IC50 value. Therefore, we selected 4T1 cells treated for 48 h with 6, 12.5, and 25 µM ISO for subsequent experiments.

### 3.2 ISO inhibited the migration of 4T1 cells

**FIGURE 2:**
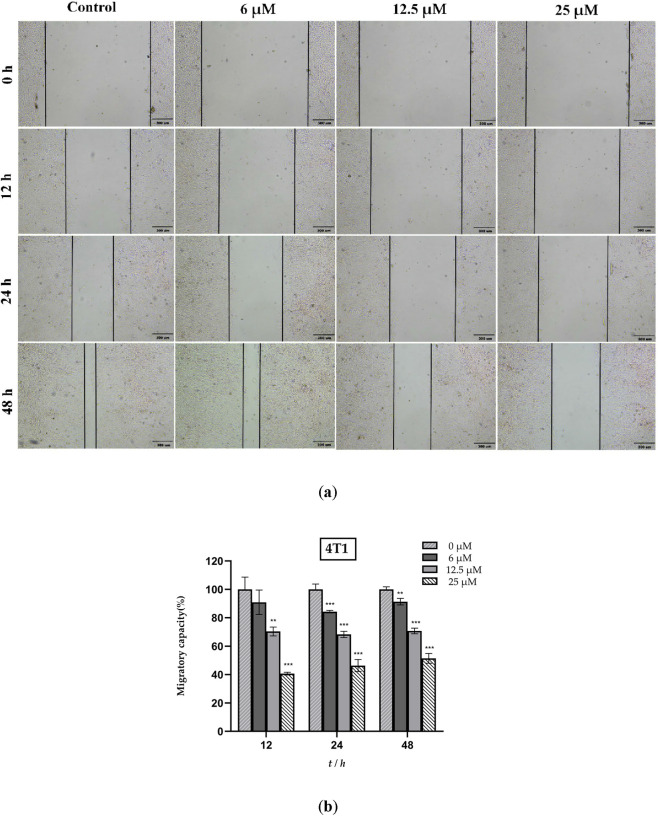
**(a)** Representative images of 4T1 cell migration after treatment with isorhamnetin (ISO) (0, 6, 12.5, and 25 μM) for 12, 24, and 48 h, assessed by wound healing assay. Untreated cells served as the control group. Scale bar = 300 μm. **(b)** Quantitative analysis of cell migration, presented as a percentage relative to the control group. Data are expressed as mean ± SD from three independent experiments (n = 3). Statistical analysis was performed using one-way ANOVA. *p < 0.05, **p < 0.01, ***p < 0.001 vs. control group.

Having established that ISO inhibited the proliferation of 4T1 cells, we further investigated its effect on cell migration. As shown in Figures 2a,b, the effects of various ISO concentrations on 4T1 cell migration were assessed using a wound healing assay. Treatment with ISO consistently reduced the area of cell migration compared with that in the control group, indicating that ISO suppressed 4T1 cell migration. Furthermore, this inhibitory effect exhibited concentration-dependent characteristics.

### 3.3 ISO inhibited the formation of 4T1 cell colonies

**FIGURE 3:**
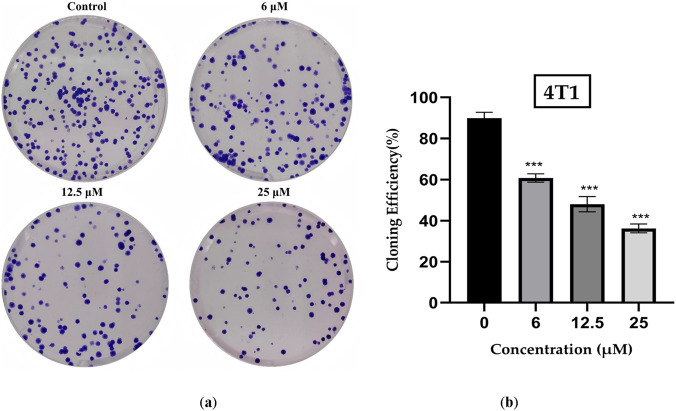
**(a)** Representative images of colony formation in 4T1 cells following treatment with isorhamnetin (ISO) (0, 6, 12.5, and 25 μM) for 48 h, assessed by colony formation assay. **(b)** Quantitative analysis of colony formation, presented as a percentage relative to the control group. Data are expressed as mean ± SD from three independent experiments (n = 3). Statistical analysis was performed using one-way ANOVA. *p < 0.05, **p < 0.01, ***p < 0.001 vs. control group.

As one of the primary markers of cellular proliferation, the colony formation assay was conducted on 4T1 cells. The results are presented in Figures 3a,b, demonstrating that various concentrations of ISO significantly reduced colony formation in 4T1 cells compared with that in the control group, exhibiting a markedly inhibitory effect on colony formation in a concentration-dependent manner. At an ISO concentration of 12.5 µM, the cell count was approximately 50% of that in the control group, whereas at 25 µM, the cell count was below 40% of that in the control group.

### 3.4 ISO promoted the generation of ROS in 4T1 cells

**FIGURE 4:**
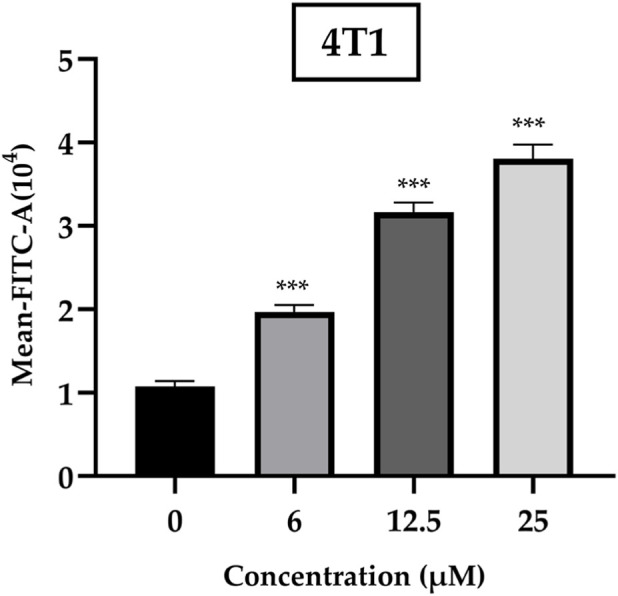
Intracellular ROS levels in 4T1 cells following treatment with isorhamnetin (ISO) (0, 6, 12.5, and 25 μM) for 48 h were analyzed by flow cytometry. Mean fluorescence intensity (FITC-A) was used to represent ROS levels. Data are expressed as mean ± SD from three independent experiments (n = 3). Statistical analysis was performed using one-way ANOVA. *p < 0.05, **p < 0.01, ***p < 0.001 vs. control group.

We employed ROS probes to investigate whether oxidative stress was involved in ISO-induced cell death in 4T1 cells. As shown in Figure 4, ISO increased ROS production in a concentration-dependent manner. At concentrations exceeding 6 μM, ROS levels were approximately two-fold higher than those in the control group, exhibiting a clear dose-dependent trend. These findings suggest that ISO-induced oxidative stress may contribute to pro-apoptotic signaling and mitochondrial dysfunction-associated cell death.

### 3.5 ISO induced mitochondrial membrane potential loss in 4T1 cells

**FIGURE 5:**
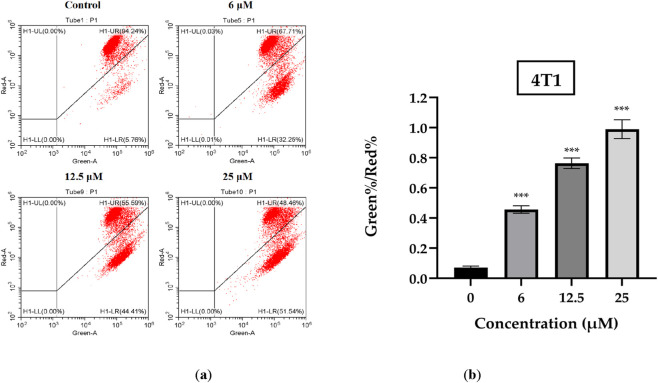
**(a)** Representative images of mitochondrial membrane potential in 4T1 cells following treatment with isorhamnetin (ISO) (0, 6, 12.5, and 25 μM) for 48 h, assessed by JC-1 staining assay. **(b)** Quantitative analysis of mitochondrial membrane potential expressed as the red/green fluorescence intensity ratio. A decrease in the red/green ratio indicates loss of mitochondrial membrane potential. Data are presented as mean ± SD from three independent experiments (n = 3). Statistical analysis was performed using one-way ANOVA. *p < 0.05, **p < 0.01, ***p < 0.001 vs. control group.

Loss of mitochondrial membrane potential is one of the hallmark events of early cellular apoptosis. We detected changes in the mitochondrial membrane potential of 4T1 cells using the JC-1 staining method. The results are shown in Figures 5a,b. The green/red fluorescence ratio of 4T1 cells treated with different concentrations of ISO detected by flow cytometry significantly increased compared with that in the control group. These findings indicated that ISO promoted the loss of mitochondrial membrane potential in 4T1 breast cancer cells in a concentration-dependent manner.

### 3.6 ISO downregulated the mRNA expression of the TIGIT/CD155 signaling axis in 4T1 cells

Numerous herbal formulations and active ingredients, such as astragalus polysaccharide and cordycepin, have been proven to regulate the tumor immune microenvironment through multi-target effects: promoting dendritic cell maturation, enhancing T/NK cell function, and even directly or indirectly regulating PD-1/PD-L1 expression. Other studies have also indicated that TCM can enhance immune checkpoint sensitivity through various mechanisms, thereby improving the efficacy of cancer immunotherapy. The TIGIT/CD155 immune checkpoint has been confirmed to inhibit the functions of NK cells and cytotoxic T cells, impede tumor cell killing, assist tumor cells in immune escape, and promote tumor growth and metastasis. Therefore, we initially identified the TIGIT/CD155 signaling axis as a potential target of ISO for further exploration.

**FIGURE 6:**
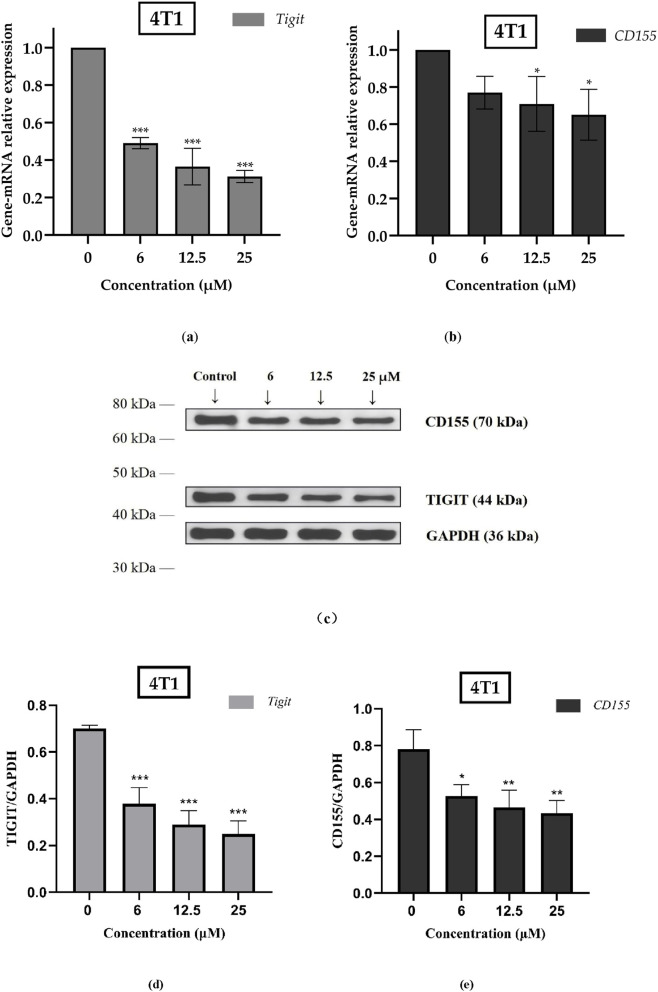
**(a,b)** mRNA analysis of TIGIT and CD155 in 4T1 cells treated with isorhamnetin (ISO) (0, 6, 12.5, and 25 µM) for 48 h. **(c)** Western blot image showing the expression of TIGIT and CD155 in 4T1 cells treated with ISO (0, 6, 12.5, and 25 µM) for 48 h. **(d)** Western blot analysis of TIGIT expression normalized to GAPDH in 4T1 cells treated with ISO (0, 6, 12.5, and 25 µM) for 48 h. **(e)** Western blot analysis of CD155 expression normalized to GAPDH in 4T1 cells treated with ISO (0, 6, 12.5, and 25 µM) for 48 h. Data are presented relative to the untreated control group and expressed as mean ± standard error (n = 3). Significance levels between the control group and experimental groups were defined as follows: *p < 0.05, **p < 0.01, ***p < 0.001.

The effect of different concentrations of ISO on the expression of the TIGIT/CD155 signaling axis was detected by quantitative real-time polymerase chain reaction (qPCR). As shown in Figures 6a,b, the mRNA level of TIGIT in 4T1 cells treated with different concentrations of ISO was significantly reduced in a concentration-dependent manner. Specifically, the expression rate in the 6 µM ISO treatment group was only about 50% of that in the untreated group. Similarly, the mRNA level of CD155 decreased in a concentration-dependent manner after ISO treatment, with the expression rate in the 12.5 µM treatment group being approximately 70% of that in the untreated group. The same trend was observed at the protein level, as shown in Figures 6c–e.

## 4 Discussion

Breast cancer is a highly heterogeneous malignant tumor characterized by high metastatic potential and low T cell immune infiltration. Therefore, inhibiting tumor cell metastasis and inducing tumor cell apoptosis are important strategies for breast cancer treatment (Zhu et al., 2023). ISO has demonstrated multiple advantages in cancer therapy in recent years, including its multi-target mechanism of action, which can simultaneously regulate multiple signaling pathways, effectively inhibit the proliferation, invasion, and metastasis of tumor cells such as pancreatic cancer, bladder cancer, and oral squamous cell carcinoma, and modulate the tumor microenvironment (Mei et al., 2025). As an immune checkpoint inhibitor, TIGIT is abnormally highly expressed in immune cells, and its binding to CD155 inhibits tumor apoptosis and promotes tumor metastasis (Zhou et al., 2018). The main mechanism is that after binding to CD155, TIGIT can inhibit the metabolic activity (e.g., glycolysis) and cytotoxic function of T cells and NK cells, leading to decreased antitumor activity (Shao et al., 2021). The binding of CD155 on tumor cells to TIGIT on tumor-infiltrating lymphocytes (TILs) inhibits T cell activation, thereby promoting tumor cell immune escape (Kawashima et al., 2021). Therefore, targeted blocking of the interaction between TIGIT and CD155 may be a promising approach to regulate tumor cell metastasis and apoptosis.

In this study, we investigated the effects of ISO on the biological behavior of 4T1 breast cancer cells and the underlying mechanisms. The results showed that ISO inhibited cell proliferation, migration, and colony formation, and may exert anti-tumor effects, potentially associated with oxidative stress and modulation of the TIGIT/CD155 signaling axis. First, ISO-induced inhibition of 4T1 cell proliferation exhibited a distinct concentration- and time-dependent manner, whereas it showed low toxicity to normal mammary epithelial HC11 cells, suggesting potential selective antitumor activity of ISO. Cell migration and colony formation assays further showed that ISO inhibited the migration and clonogenic capacity of 4T1 cells, suggesting its potential role in suppressing breast cancer cell progression. The mechanistic exploration revealed that ISO treatment led to an increase in ROS levels and a decrease in mitochondrial membrane potential in 4T1 cells, suggesting that ISO may trigger oxidative stress and mitochondrial dysfunction, potentially contributing to the induction of apoptosis. This finding is consistent with the common mechanisms observed in the antitumor effects of various natural products (Sharifi-Rad et al., 2021; Yang et al., 2022; Wu et al., 2023; Farhoudi et al., 2024). Furthermore, this study demonstrates that isorhamnetin (ISO) downregulates the mRNA expression of the TIGIT/CD155 immune checkpoint axis, suggesting a potential role in enhancing anti-tumor immune responses. Given that the TIGIT/CD155 pathway is known to suppress the activity of NK cells and cytotoxic T lymphocytes, its downregulation may contribute to the restoration of immune cell-mediated tumor killing and inhibition of tumor immune escape.

The underlying mechanisms by which isorhamnetin regulates TIGIT/CD155 expression may involve multiple signaling pathways. Previous studies have shown that isorhamnetin can modulate key pathways associated with tumor progression and immune regulation, including NF-κB, PI3K/AKT, and MAPK signaling. These pathways are closely linked to the regulation of immune checkpoint molecules; thus, their inhibition may lead to reduced TIGIT/CD155 expression.

In addition, oxidative stress appears to play an important intermediary role. In the present study, isorhamnetin significantly altered intracellular ROS levels, which are known to regulate signaling pathways such as NF-κB and MAPK. Therefore, modulation of ROS by isorhamnetin may indirectly influence TIGIT/CD155 expression through these downstream pathways.

Moreover, mitochondrial dysfunction and apoptosis induced by isorhamnetin, as indicated by changes in mitochondrial membrane potential, may further contribute to this regulatory process. These cellular events are closely associated with transcriptional regulation mediated by factors such as NF-κB, STAT3, and HIF-1α, which are known to participate in immune modulation and tumor progression.

However, the precise molecular mechanisms underlying these effects require further investigation.

It should be noted that our findings regarding TIGIT and CD155 were observed directly in 4T1 tumor cells. In addition, functional assays in this study were primarily conducted in 4T1 cells without parallel validation in normal mammary epithelial cells such as HC11. Although the proliferation assay provided preliminary evidence of differential responses between malignant and normal-like cells, further studies are required to comprehensively evaluate the selectivity and safety profile of isorhamnetin.

While these molecules are key players in the immune checkpoint pathway, the absence of immune cell involvement in our current experimental setup means that the functional impact on the immune microenvironment remains speculative. Future studies employing co-culture systems or immunocompetent animal models are warranted to confirm the immunomodulatory effects of isorhamnetin. In addition, the modified scratch assay design involving pretreatment prior to reseeding may influence the interpretation of migration results, and the absence of invasion assays limits conclusions regarding metastatic potential.

In summary, isorhamnetin (ISO) inhibited the proliferation and migration of 4T1 breast cancer cells, accompanied by increased oxidative stress and mitochondrial dysfunction. Notably, ISO downregulated the expression of the TIGIT/CD155 immune checkpoint signaling axis, suggesting a potential role in modulating tumor immune escape. These findings indicate that ISO may exert its anti-tumor effects through coordinated regulation of oxidative stress, mitochondrial function, and immune checkpoint signaling. This study provides new insights into the immunomodulatory mechanisms of ISO and highlights its potential as a candidate agent for breast cancer therapy.

## Data Availability

The original contributions presented in the study are included in the article/Supplementary Material, further inquiries can be directed to the corresponding author.
