# Supplementary material for: Isorhamnetin inhibits migration and promotes apoptosis via modulation of oxidative stress, mitochondrial dysfunction, and the TIGIT/CD155 axis in 4T1 breast cancer cells
Source: Front Cell Dev Biol. 2026 May 7;14:1829358. doi: 10.3389/fcell.2026.1829358 (PMC13189752; doi:10.3389/fcell.2026.1829358)
Supplement: Supplementary file 1 [file DataSheet1.docx]

Supplementary Material

# Supplementary Figures and Tables

**1.1 Table 1.** Primers used in the study

| **Name** | **Primer** | **Sequence** | **Size (bp)** |
| --- | --- | --- | --- |
| Mus *GAPDH* | Forward | ATGGGTGTGAACCACGAGA | 229 |
|  | Reverse | CAGGGATGATGTTCTGGGCA |  |
| Mus *TIGIT* | Forward | TGGGACTCATTTGCTTAATGGT | 164 |
|  | Reverse | AGTTTGTGTCTGGACAGGGCTT |  |
| Mus *CD155* | Forward | CTGCTGTTCTGCTATGCACTCC | 232 |
|  | Reverse | CCCTCTCTGGCTCTTTGATGTT |  |

## Supplementary Figures


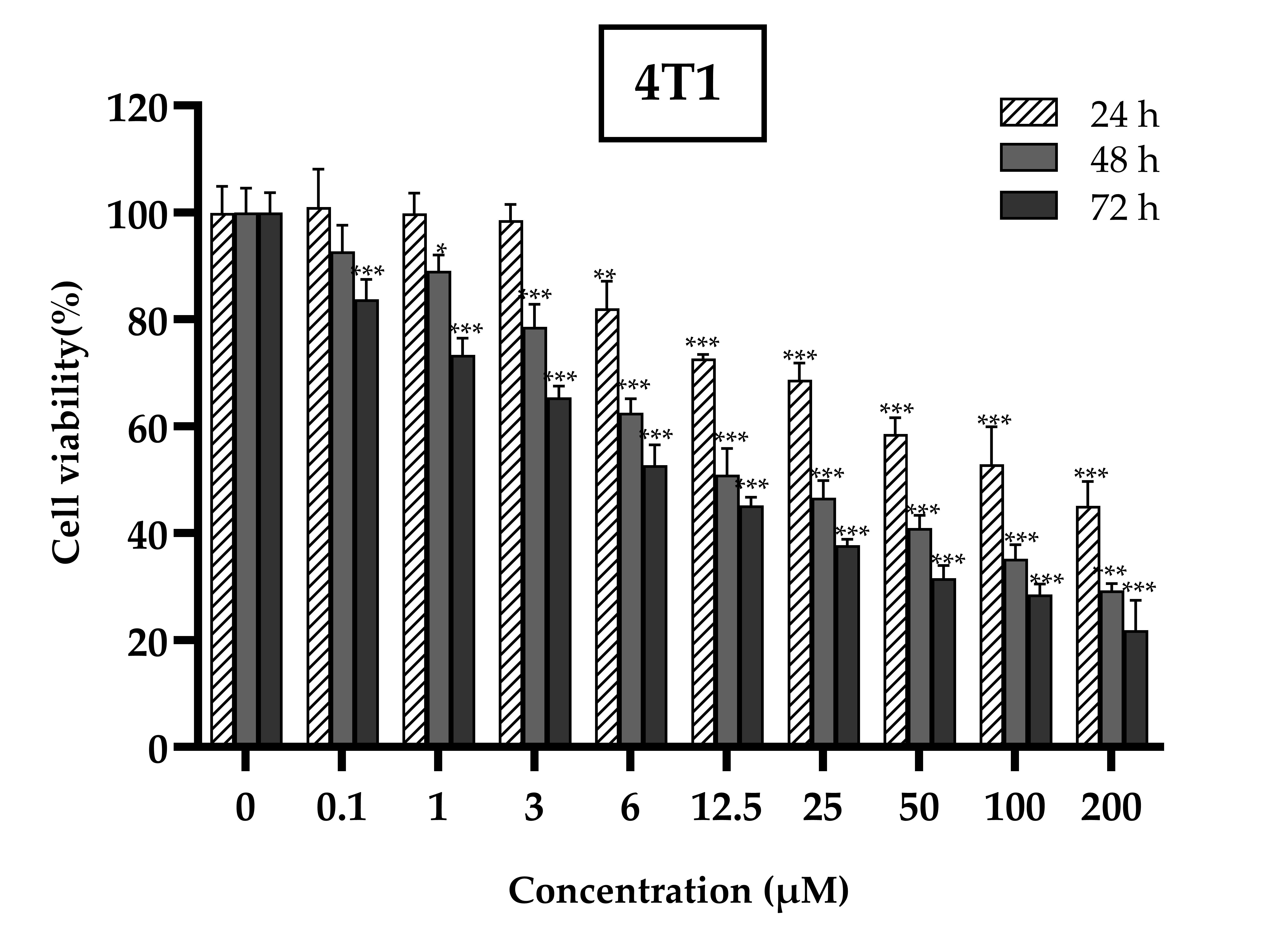

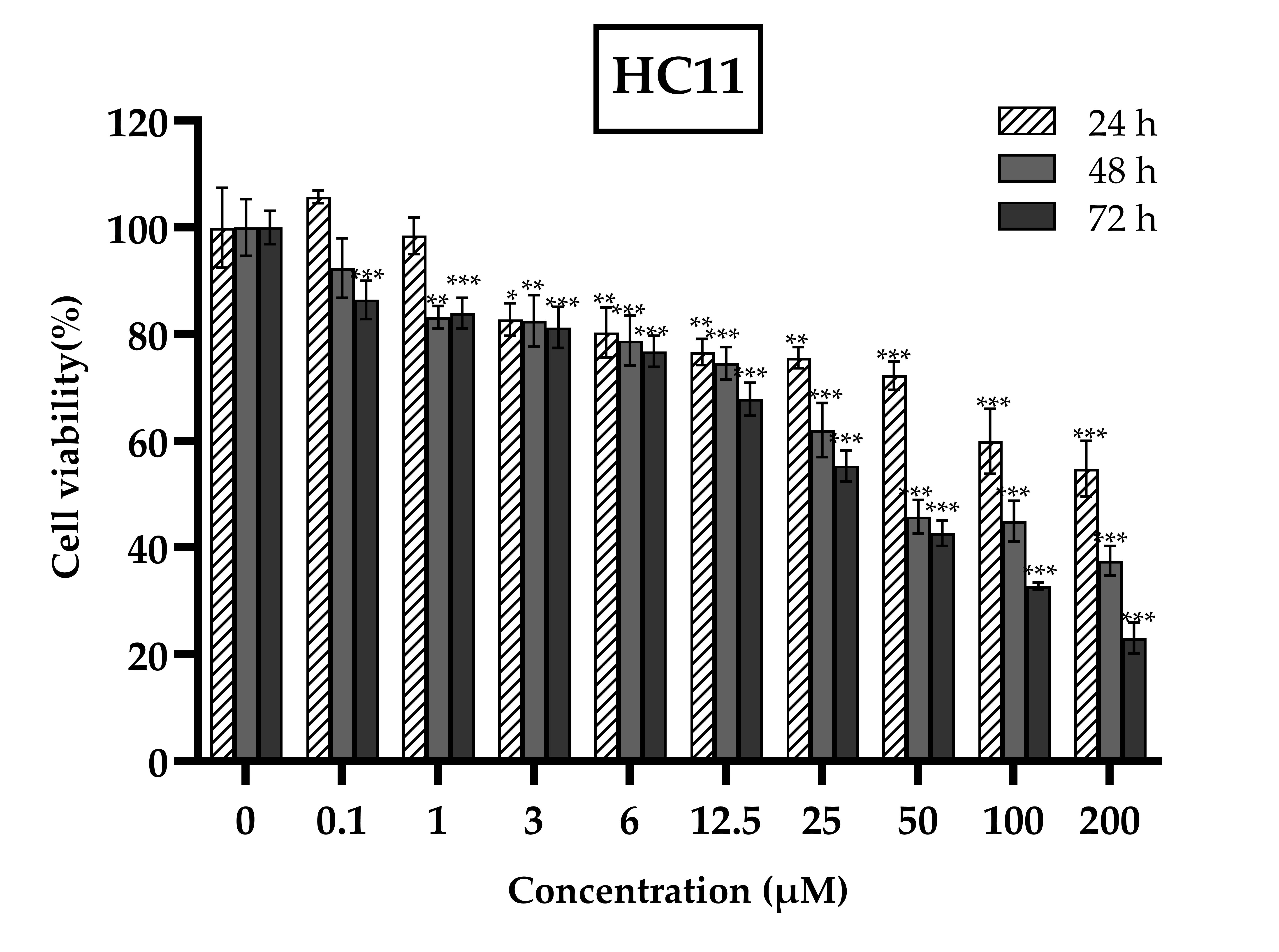


| (**a**) | (**b**) |
| --- | --- |

**Figure 1.** Mouse 4T1 mammary carcinoma cells **(a)** and mouse HC11 mammary epithelial cells **(b)** were seeded into 96-well plates and treated with isorhamnetin (ISO) at indicated concentrations for 24, 48, and 72 h. Cell proliferation was assessed using the CCK-8 assay. Data are presented as mean ± SD from three independent experiments (n = 3). Statistical analysis was performed using one-way ANOVA. **p* < 0.05, ***p* < 0.01, ****p* < 0.001 vs. control group.


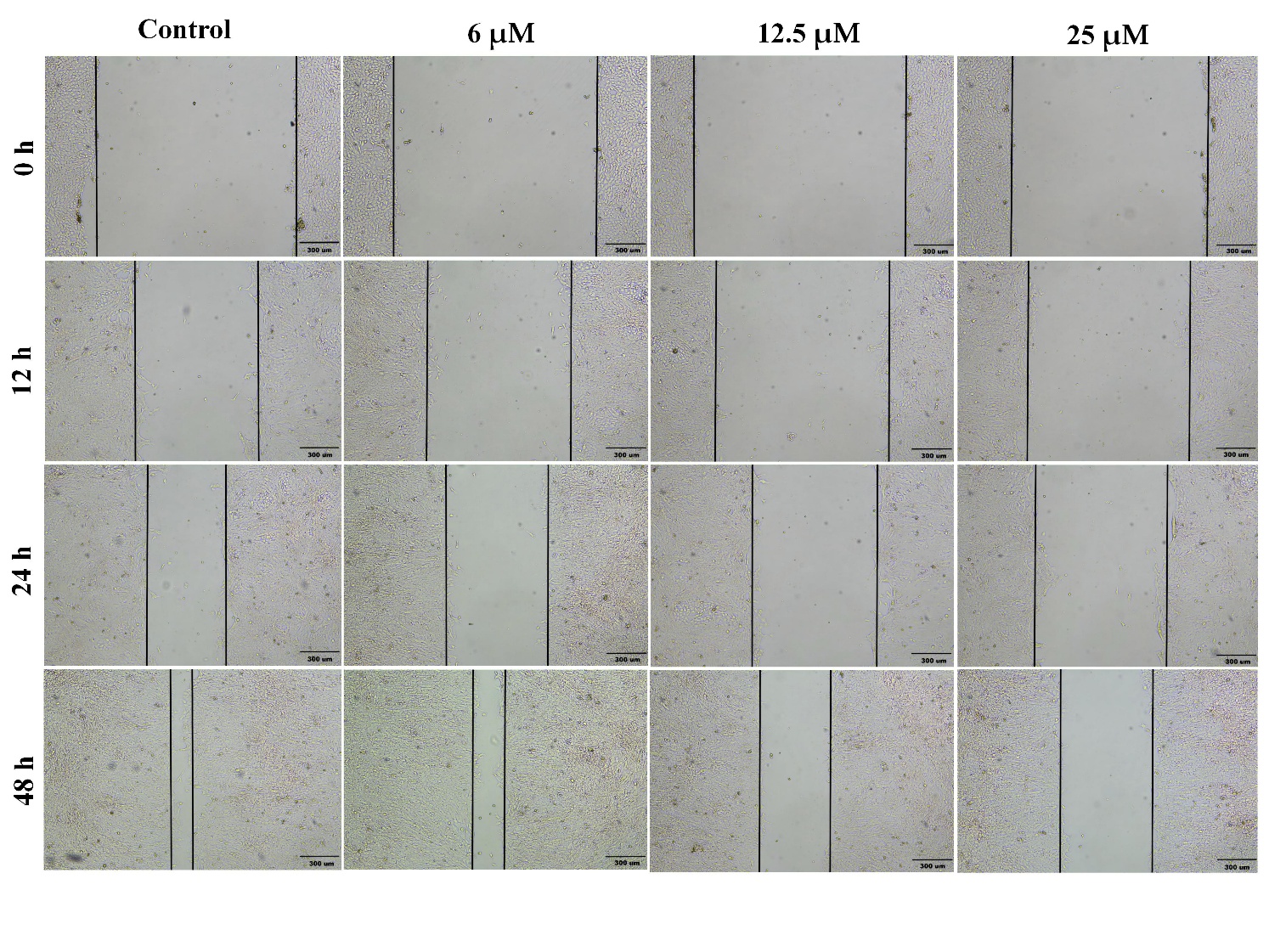


(**a**)


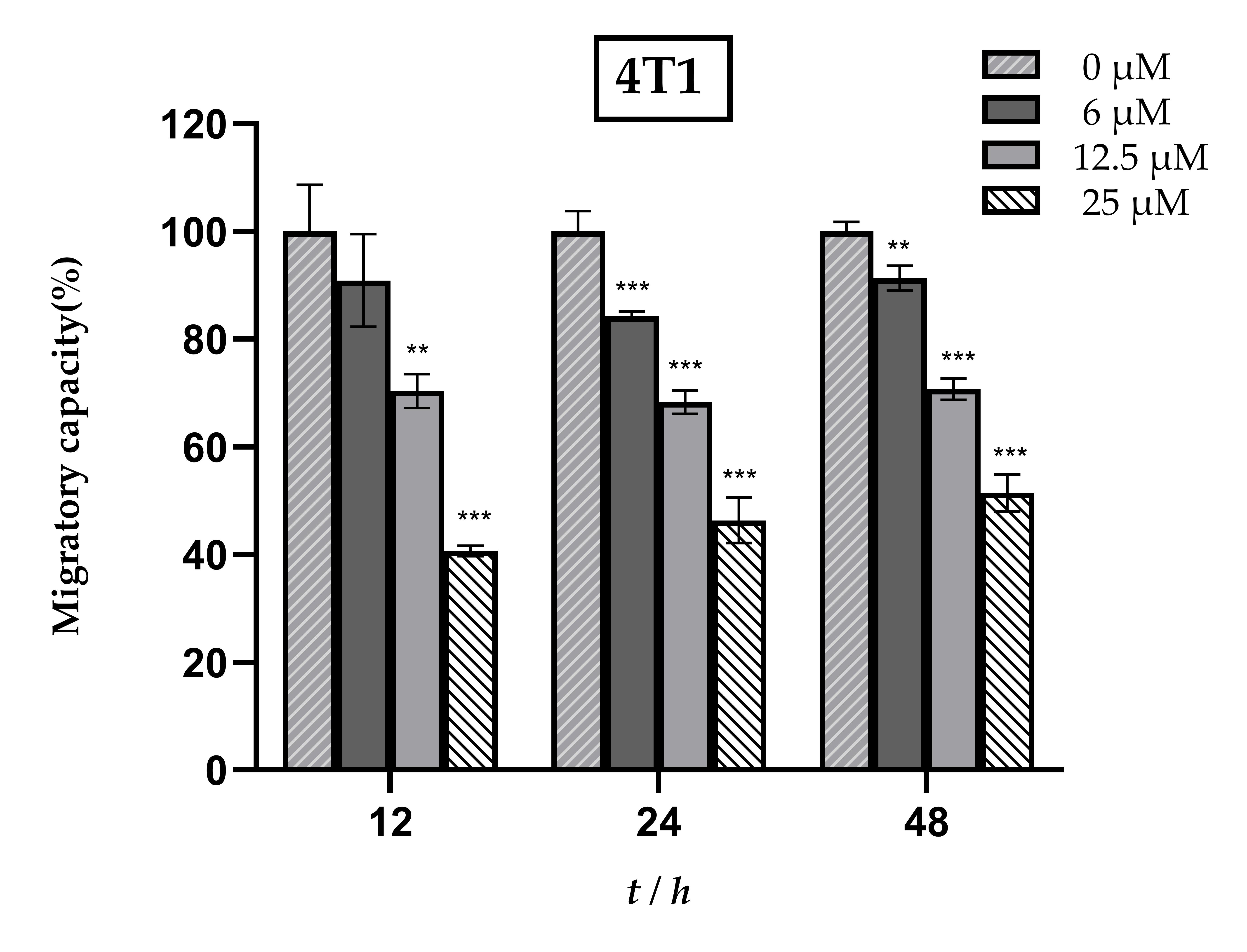


(**b**)

**Figure 2.** **(a)** Representative images of 4T1 cell migration after treatment with isorhamnetin (ISO) (0, 6, 12.5, and 25 μM) for 12, 24, and 48 h, assessed by wound healing assay. Untreated cells served as the control group. Scale bar = 300 μm. **(b)** Quantitative analysis of cell migration, presented as a percentage relative to the control group. Data are expressed as mean ± SD from three independent experiments (n = 3). Statistical analysis was performed using one-way ANOVA. **p* < 0.05, ***p* < 0.01, ****p* < 0.001 vs. control group.


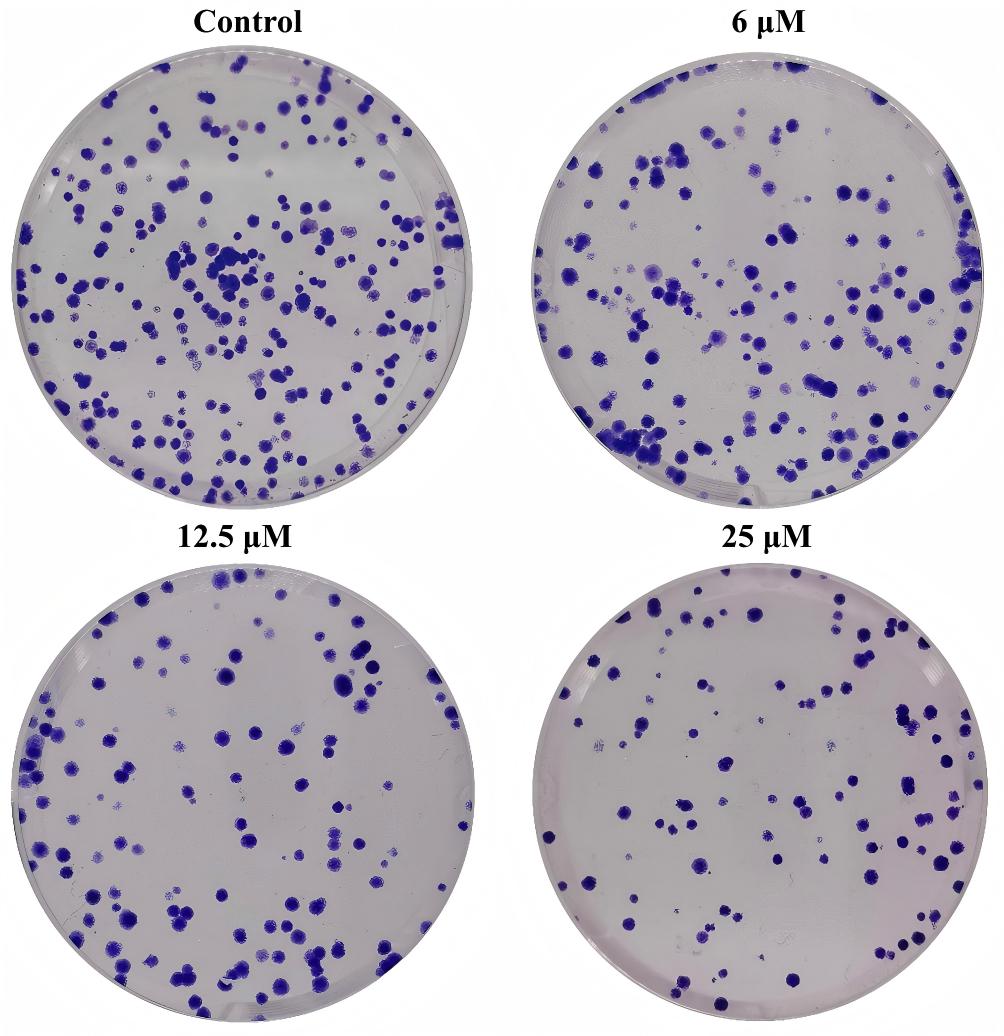

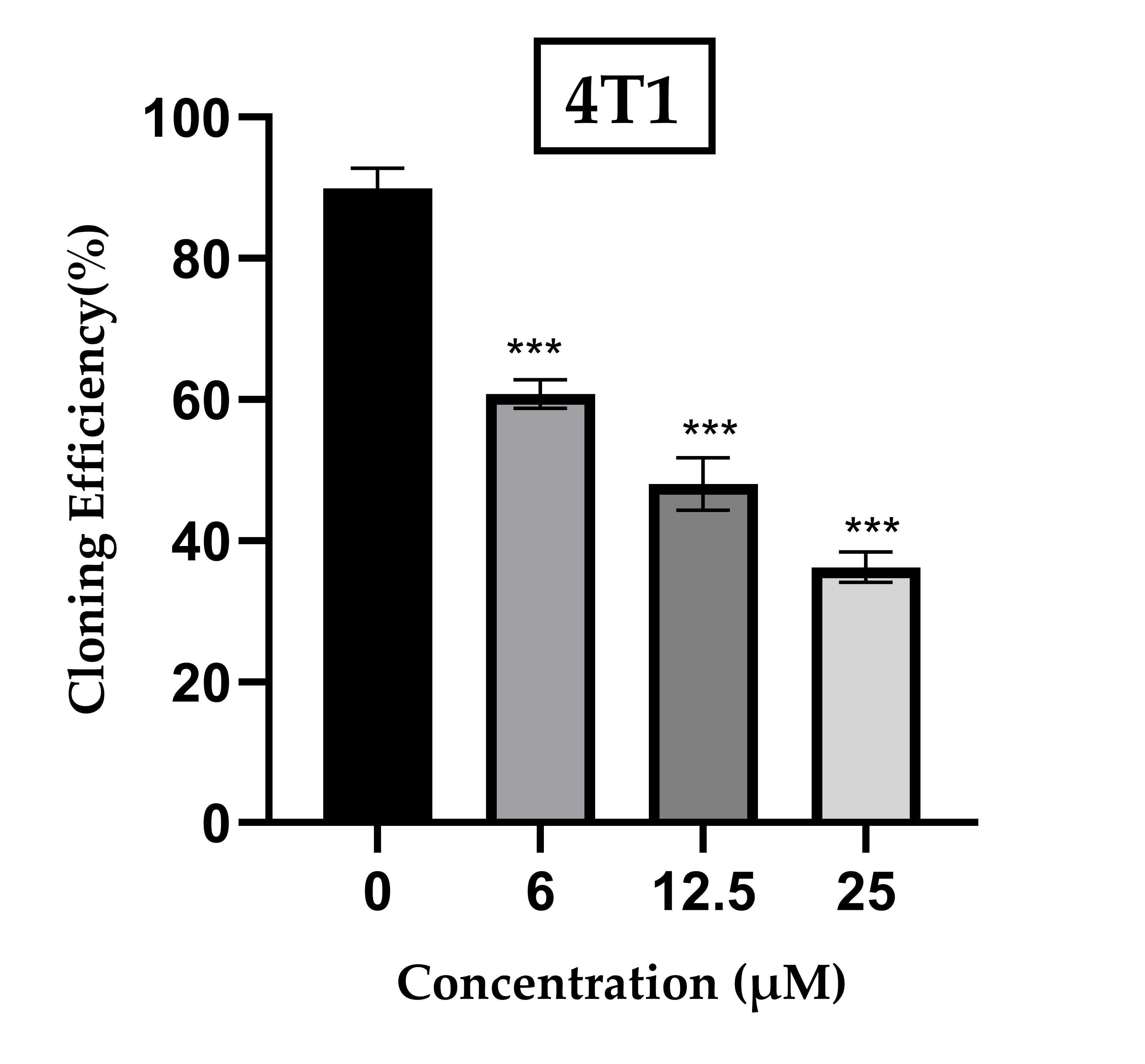


| (**a**) | (**b**) |
| --- | --- |

**Figure 3. (a)** Representative images of colony formation in 4T1 cells following treatment with isorhamnetin (ISO) (0, 6, 12.5, and 25 μM) for 48 h, assessed by colony formation assay. **(b)** Quantitative analysis of colony formation, presented as a percentage relative to the control group. Data are expressed as mean ± SD from three independent experiments (n = 3). Statistical analysis was performed using one-way ANOVA. **p* < 0.05, ***p* < 0.01, ****p* < 0.001 vs. control group.

**
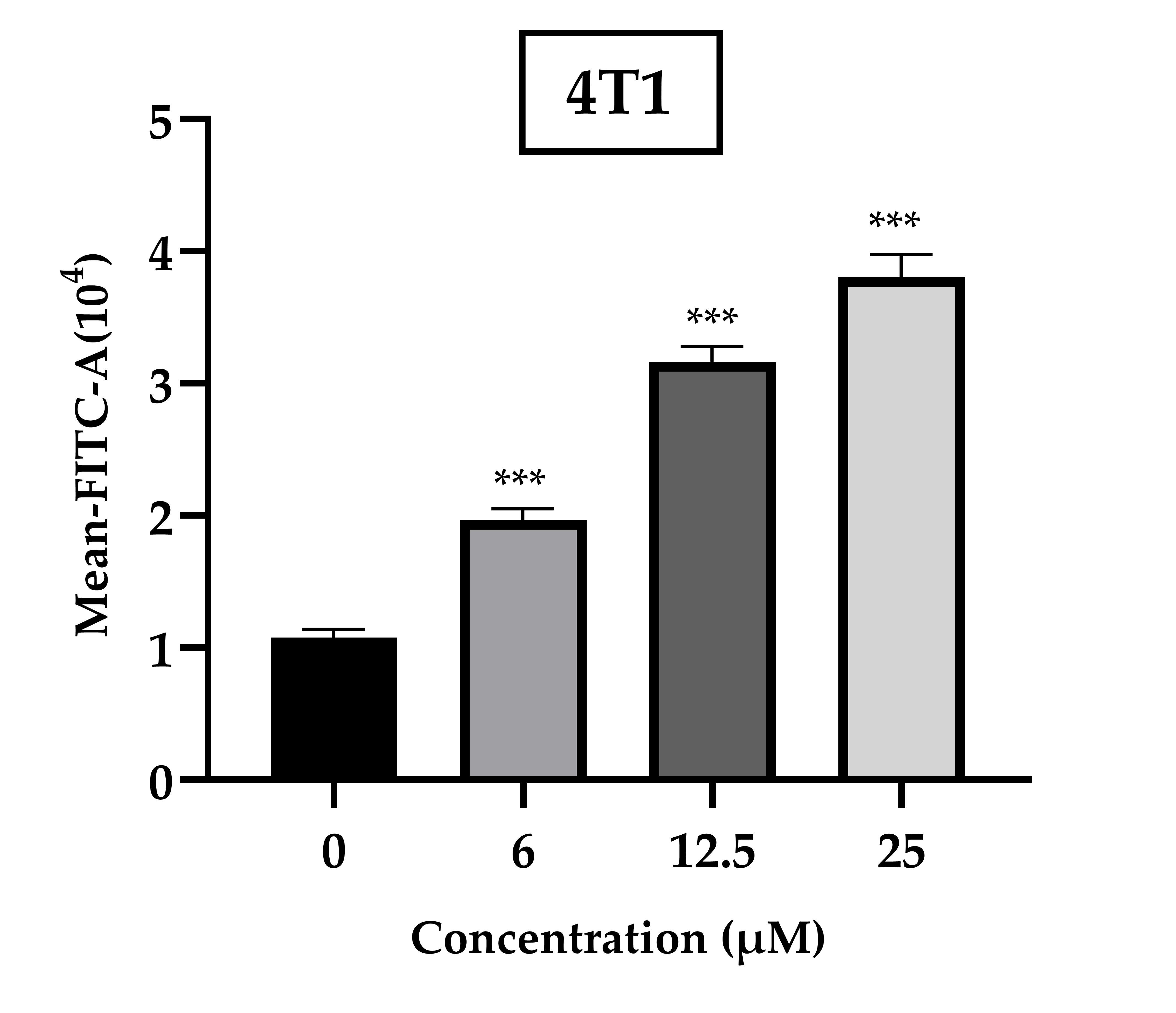
**

**Figure 4.** Intracellular ROS levels in 4T1 cells following treatment with isorhamnetin (ISO) (0, 6, 12.5, and 25 μM) for 48 h were analyzed by flow cytometry. Mean fluorescence intensity (FITC-A) was used to represent ROS levels. Data are expressed as mean ± SD from three independent experiments (n = 3). Statistical analysis was performed using one-way ANOVA. **p* < 0.05, ***p* < 0.01, ****p* < 0.001 vs. control group.


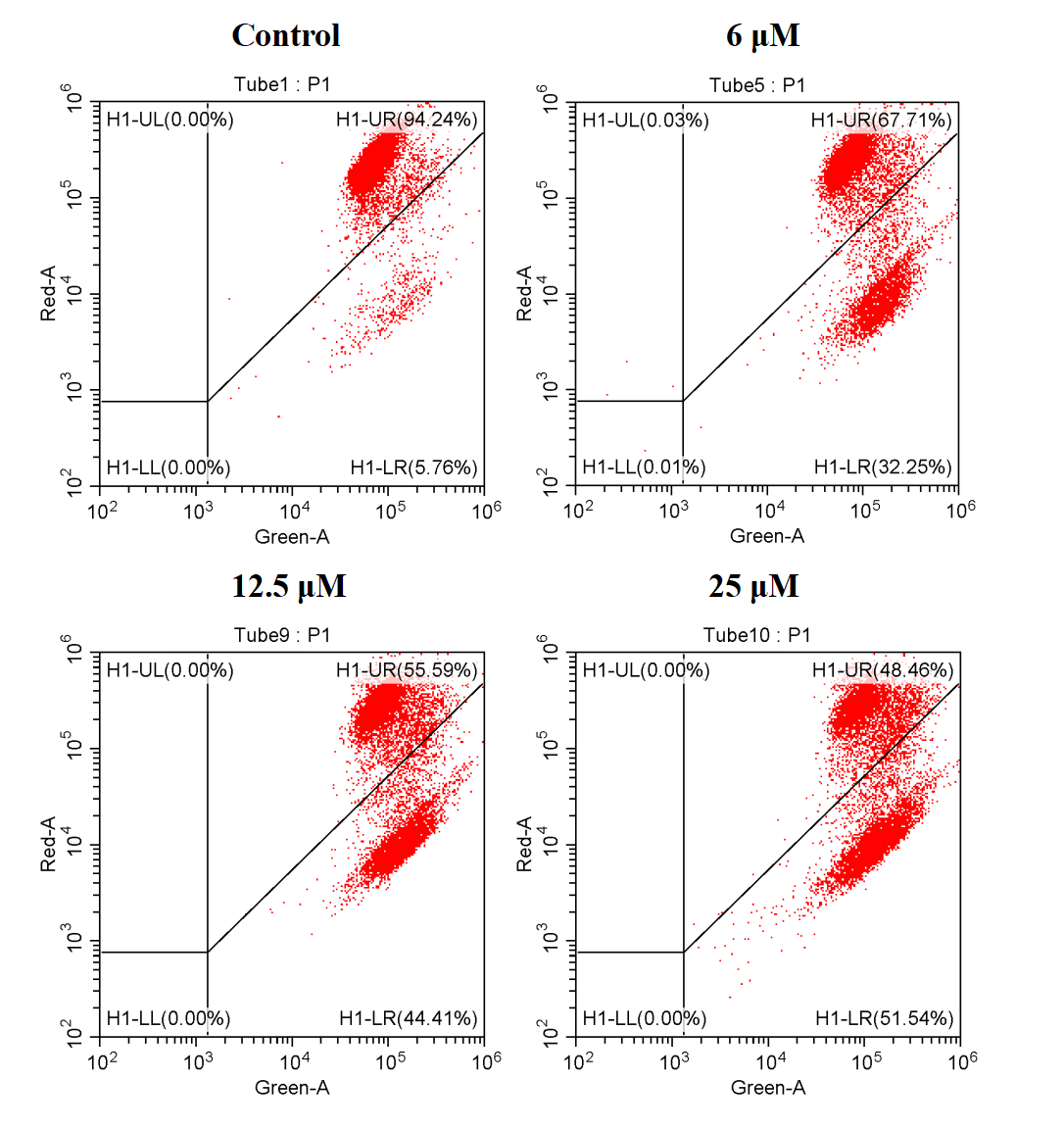

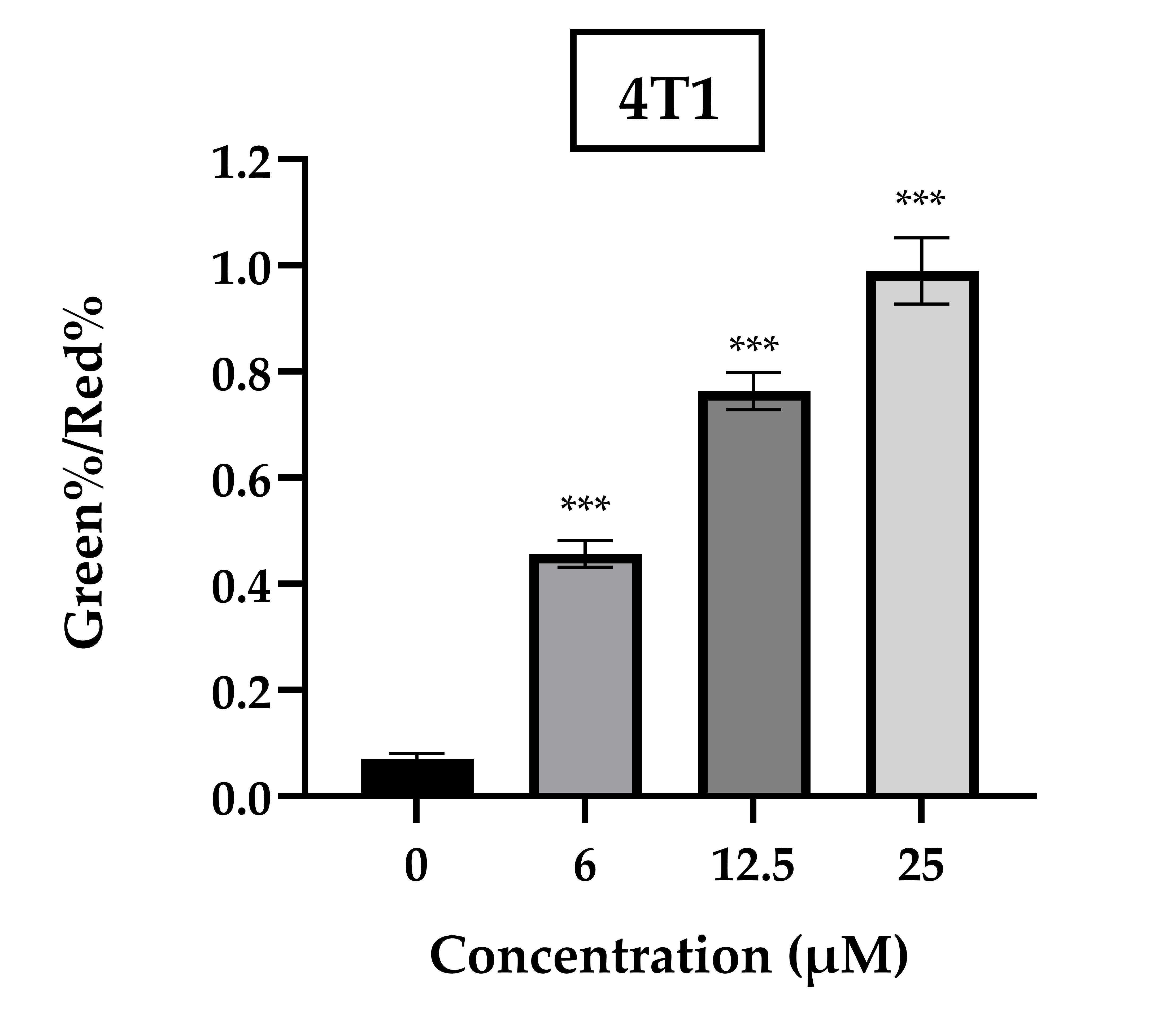


| (**a**) | (**b**) |
| --- | --- |

**Figure 5. (a)** Representative images of mitochondrial membrane potential in 4T1 cells following treatment with isorhamnetin (ISO) (0, 6, 12.5, and 25 μM) for 48 h, assessed by JC-1 staining assay. **(b)** Quantitative analysis of mitochondrial membrane potential expressed as the red/green fluorescence intensity ratio. A decrease in the red/green ratio indicates loss of mitochondrial membrane potential. Data are presented as mean ± SD from three independent experiments (n = 3). Statistical analysis was performed using one-way ANOVA. **p* < 0.05, ***p* < 0.01, ****p* < 0.001 vs. control group.


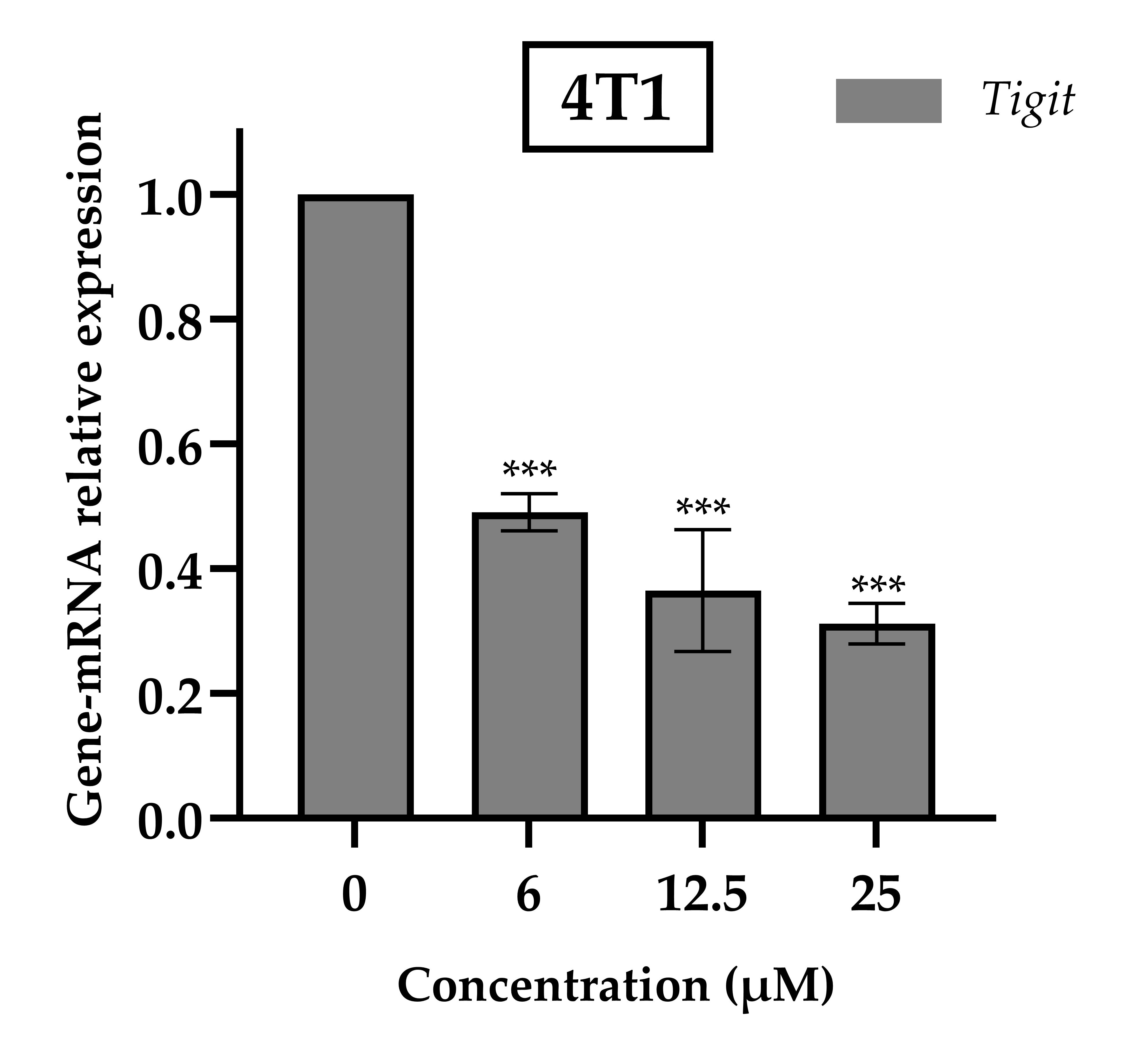

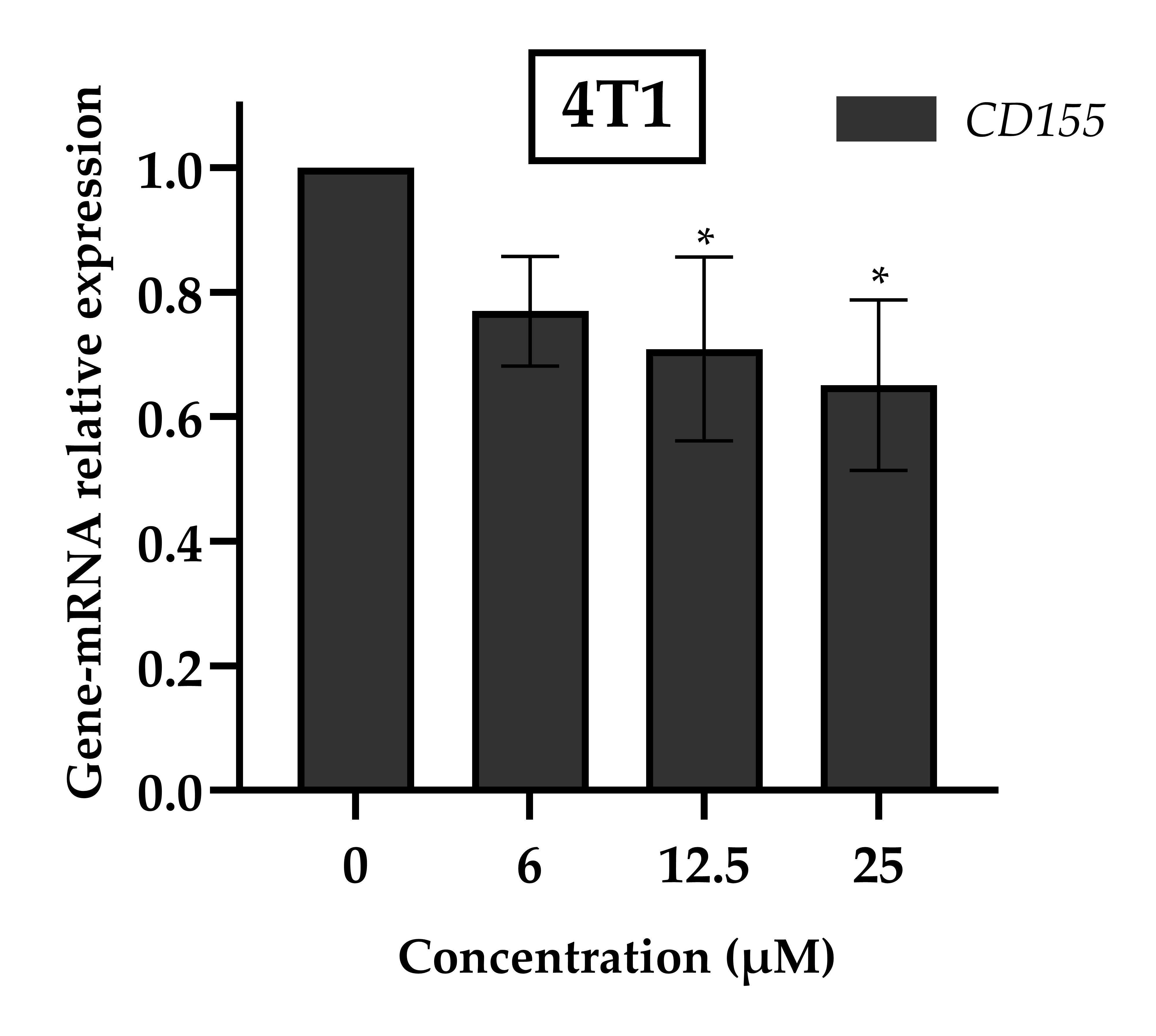


| (**a**) | (**b**) |
| --- | --- |


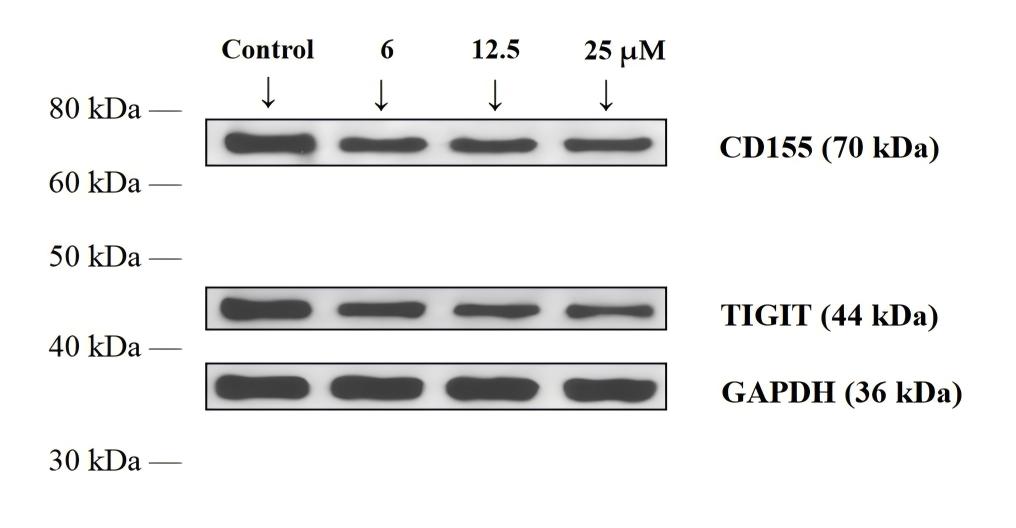


**（c）**


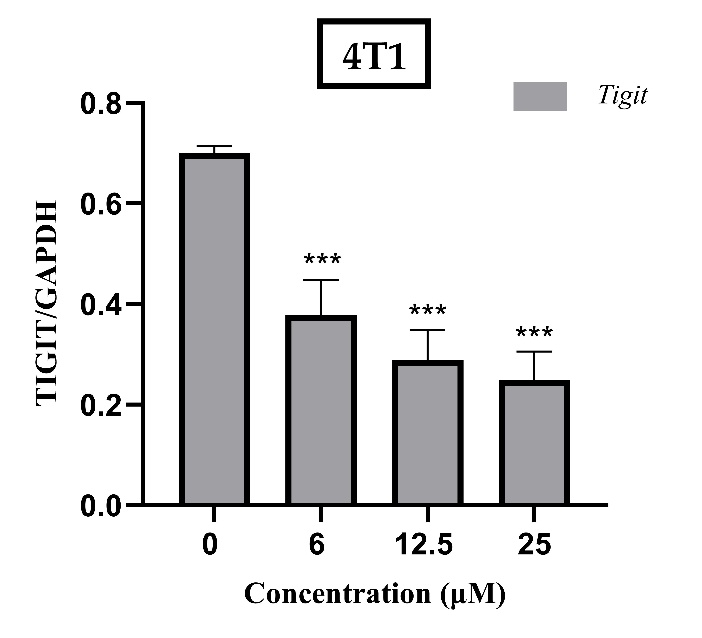
**
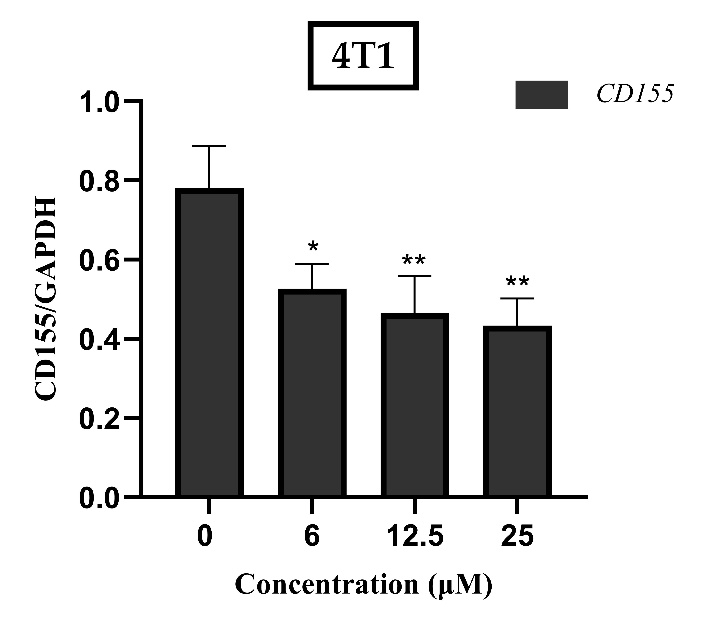
**

**(d) (e)**

**Figure 6.** **(a, b)** mRNA analysis of TIGIT and CD155 in 4T1 cells treated with isorhamnetin (ISO) (0, 6, 12.5, and 25 µM) for 48 h. **(c)** Western blot image showing the expression of TIGIT and CD155 in 4T1 cells treated with ISO (0, 6, 12.5, and 25 µM) for 48 h. **(d)** Western blot analysis of TIGIT expression normalized to GAPDH in 4T1 cells treated with ISO (0, 6, 12.5, and 25 µM) for 48 h. **(e)** Western blot analysis of CD155 expression normalized to GAPDH in 4T1 cells treated with ISO (0, 6, 12.5, and 25 µM) for 48 h. Data are presented relative to the untreated control group and expressed as mean ± standard error (n = 3). Significance levels between the control group and experimental groups were defined as follows: **p* < 0.05, ***p* < 0.01, ****p* < 0.001.
